# Not Just in the Mind: A Case of Neurological Decline in a Young Adult With Psychiatric History

**DOI:** 10.7759/cureus.90929

**Published:** 2025-08-25

**Authors:** Neoma Lemos

**Affiliations:** 1 Emergency Medicine, Queen's Hospital, Barking, Havering and Redbridge University Hospitals NHS Trust, London, GBR

**Keywords:** ct and mri brain, diagnostic overshadowing, gait ataxia, neurology case report, psychiatric comorbidity, relapsing-remitting multiple sclerosis, schizophrenia, specific learning disability

## Abstract

A 35-year-old man with a background of schizophrenia and mild learning disability presented to the emergency department with a four-day history of left-sided numbness and fine motor difficulties, describing his hand as “not getting signals from his brain.” Over the preceding few months, he had developed progressive unsteadiness, frequent falls, and significant weight loss. Despite these neurologic red flags, his psychiatric history initially skewed clinical suspicion toward a functional or psychogenic cause. On assessment, he was alert but mildly disoriented to time. Neurological examination revealed left-sided pronator drift, impaired coordination, and subtle upper limb weakness. Cranial nerves were intact, with no dysarthria or nystagmus. Although the initial computed tomography (CT) scan and angiography of the brain and its blood vessels were unremarkable, the persistence and evolution of his symptoms prompted escalation. A magnetic resonance imaging (MRI) of the brain revealed multiple partly confluent white matter lesions affecting the cerebral hemispheres, cerebellar peduncles, and brainstem, findings consistent with a demyelinating process. Neurology review confirmed a diagnosis of relapsing-remitting multiple sclerosis (MS). As the relapse was mild, corticosteroids were not initiated. He was discharged with a referral to the MS multidisciplinary team for long-term management. This case highlights the risk of diagnostic overshadowing, where psychiatric comorbidity diverts attention from organic pathology. It demonstrates the importance of pursuing definitive imaging when clinical suspicion remains high despite normal initial studies, with MRI proving decisive in establishing the diagnosis. The case also highlights the importance of listening to family, maintaining a diagnostic curiosity, and resisting cognitive biases. Clinicians must remain alert to the possibility of serious neurological disease, even when the patient’s history seems to suggest otherwise.

## Introduction

Multiple sclerosis (MS) is a chronic autoimmune demyelinating disease of the central nervous system (CNS) and is a leading cause of non-traumatic neurological disability in young adults, particularly women, and often presents between the ages of 20 and 40 [1]. The disease course can be highly variable, and common presenting symptoms include sensory disturbances, visual changes, motor weakness, and gait imbalance. In MS, diagnosis relies on demonstrating "dissemination in time and space," meaning evidence of inflammatory damage appearing in different parts of the central nervous system at separate points in time, typically confirmed by magnetic resonance imaging (MRI) [2]. Diagnosing MS in individuals with coexisting psychiatric conditions presents a unique clinical challenge. The presence of mental health diagnoses can influence decision-making, particularly in emergency settings, through a phenomenon known as diagnostic overshadowing - where new or worsening neurological symptoms are misattributed to an existing psychiatric disorder such as schizophrenia, depression, anxiety, or dissociative identity disorder (DID), which can result in delayed or missed recognition of underlying medical conditions [3,4]. In particular, the overlap between psychiatric disorders such as schizophrenia and MS - including shared features like psychotic symptoms, cognitive changes, and similar age of onset - can further complicate timely recognition.

Emergency department (ED) clinicians are more susceptible to the phenomenon of "diagnostic overshadowing" due to time constraints, frequent high acuity, and limited access to collateral information [5]. Psychiatric comorbidities are common in the general population and are also increasingly being recognized among patients with MS [6], with the potential to mimic or exacerbate neurological symptoms and further complicate the diagnostic process [7]. The literature consistently shows that individuals with psychiatric diagnoses experience longer diagnostic delays, lower referral rates for imaging, and less aggressive investigation of new neurological symptoms [8,9]. In this case, although the patient had a psychiatric history, the team appropriately considered a neurological differential early, which highlights both the importance of vigilance against overshadowing and the value of structured assessment in avoiding misattribution. This case illustrates how persistent focal neurological signs, combined with timely collateral history from family, prompted further investigation despite normal initial imaging - leading to the correct diagnosis of MS.

## Case presentation

Atypical neurological complaints in a psychiatric patient 

A 35-year-old man with a background of schizophrenia, multiple personality disorder, and mild learning disability presented to the ED with a four-day history of progressive numbness affecting the left hand. He described difficulty performing fine motor tasks, such as turning a key, and expressed concern that his hand was “not getting signals from his brain.” Over the preceding two months, he had experienced increasing unsteadiness and frequent falls. He was on regular antipsychotic medications, and medication-related side effects were considered but felt unlikely given the symptom pattern. Although he had a longstanding psychiatric history, his new focal neurological symptoms were not typical of his baseline condition and therefore raised concern for an alternative diagnosis. In addition, his mother reported that he had lost approximately five stone in body weight over the past six months. This significant weight loss raised concern for alternative diagnoses such as malignancy, metabolic disease, or chronic infection, although these were considered less likely as the presentation evolved.

Collateral history revealing functional decline 

The patient was accompanied by his mother, who provided a detailed collateral history. She noted a clear decline in his physical functioning, reduced engagement with daily activities, and progressive difficulty mobilising. There were no reported medication changes, no signs of acute psychosis, and no recent hospital admissions. She emphasised that the recent changes were out of keeping with his baseline mental state and functional ability. 

ED assessment and initial impression 

On arrival at the ED, his vital signs were stable, with normal temperature, heart rate, blood pressure, and oxygen saturation. He was alert, cooperative, and oriented to person and place, although mildly disoriented to time. His disorientation to time was likely due to a combination of factors, including his mild learning disability and the prolonged ED wait in an environment without natural light. Neurological examination revealed mild left-sided upper limb weakness graded as 4 out of 5 on the Medical Research Council (MRC) scale, as well as pronator drift and impaired coordination on finger-to-nose testing, consistent with features of a mild pyramidal syndrome. Strength in the lower extremities was intact. There were no cranial nerve deficits, no dysarthria, no nystagmus, no abnormal reflexes, and no objective sensory loss noted. Tone was normal throughout, and plantar responses were flexor bilaterally. His gait was notably unsteady, consistent with a mild pyramidal pattern, but he remained ambulatory without the use of aids. The initial NIHSS score was 2. Given the presence of focal neurological signs and his elevated vascular risk factors, including a body mass index (BMI) over 30, a cholesterol level of 6.2 mmol/L, and antipsychotic use, an ischemic stroke was considered likely. The stroke team was consulted for further evaluation. A summary of key milestones from initial presentation to diagnosis and discharge is provided in Table 1.

**Table 1:** Timeline of clinical events from emergency presentation to diagnosis of multiple sclerosis UTC: Urgent Treatment Centre, ED: Emergency Department, CT: computed tomography, MRI: magnetic resonance imaging, majors: refers to the section of the ED where patients with higher-acuity needs (e.g., requiring closer monitoring or urgent investigations) are managed. This timeline outlines key milestones from initial presentation, including presenting symptoms, collateral history, examination findings, investigations, and key delays (notably a 10-hour wait to ED review due to departmental crowding), through to specialist input and confirmed diagnosis of relapsing-remitting multiple sclerosis.

Day	Time	Event
Day 1	14:30	Presented to UTC with focal weakness + unsteadiness, with his mother
Day 1	15:45	Initial assessment, bloods, CT head; triaged to Majors due to focal neurology + psychiatric background
Day 2	00:45	ED review confirmed focal neurology (delayed due to departmental crowding)
Day 2	04:00	CT head and angiogram - Unconclusive, labs unremarkable; persisting neuro deficit
Day 2	07:30	Stroke Team assessed; MRI arranged
Day 2	10:30	MRI Brain – Demyelinating lesions
Day 2	11:30	Neurology Team confirmed diagnosis of multiple sclerosis sensory relapse, discharge plan with OPD follow-up

Escalation and diagnostic imaging 

**Figure 1:**
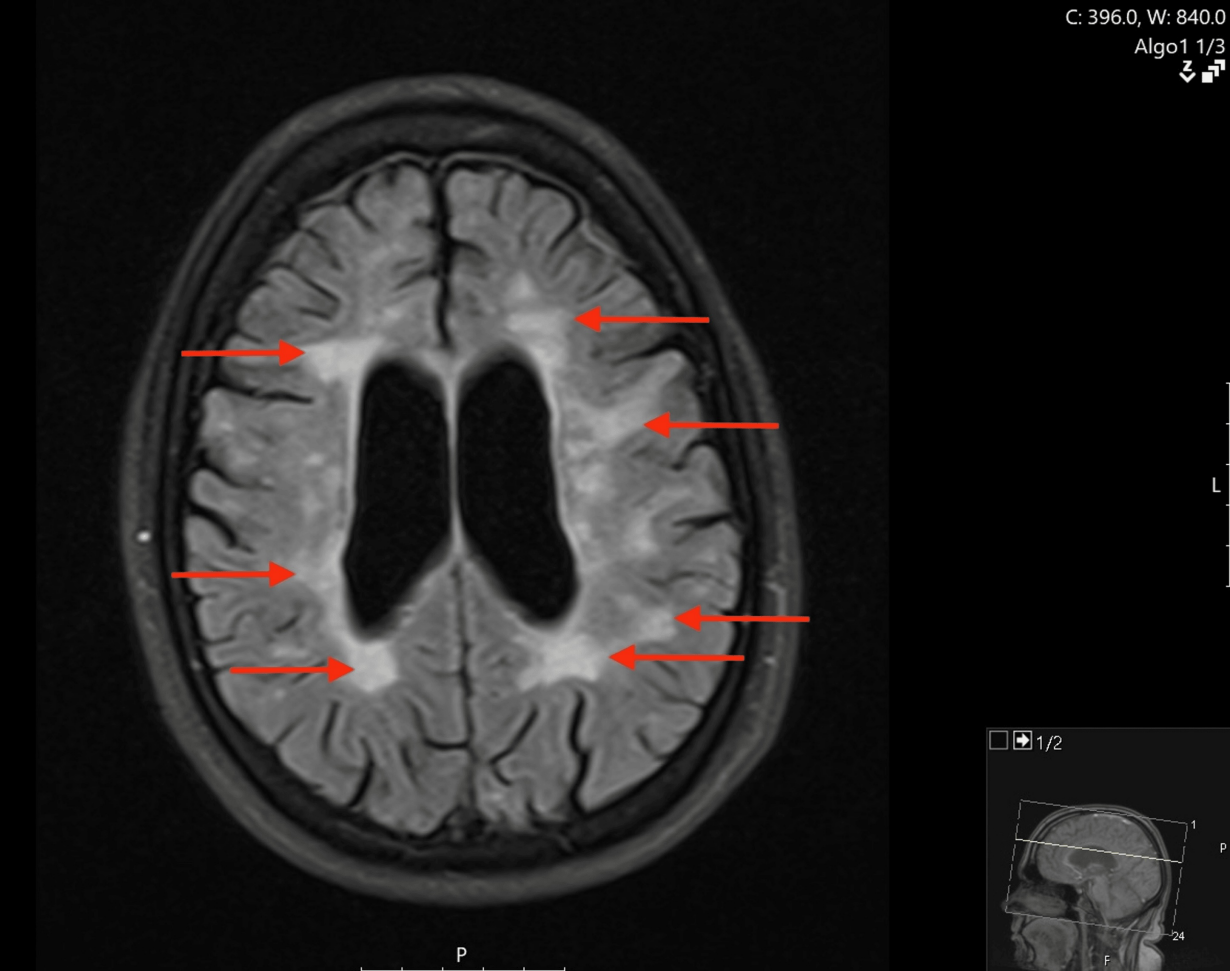
Axial and sagittal T2-weighted MRI images with gadolinium contrast demonstrating multifocal white matter lesions T2-weighted MRI sequences with contrast showing multiple partly confluent foci of high signal intensity in the cerebral hemispheres, brainstem, and cerebellar peduncles (red arrows). Some lesions demonstrate contrast enhancement while others do not, consistent with dissemination in time. The distribution of lesions demonstrates dissemination in space, supporting a diagnosis of relapsing–remitting multiple sclerosis in the clinical context.

Initial imaging included a CT head and CT angiogram, both of which were unremarkable with no evidence of acute infarction or vascular occlusion. Routine blood tests, including full blood count, renal function, liver function, and inflammatory markers, were all within normal limits apart from an elevated cholesterol level of 6.2. Despite the absence of CT abnormalities, the ongoing and progressive nature of the patient’s symptoms prompted escalation to MRI. An MRI of the brain was performed on day 2 of admission, with gadolinium contrast administered, which revealed multiple partly confluent foci of high signal intensity on T2-weighted imaging involving the cerebral hemispheres, cerebellar peduncles, and brainstem, with lesions showing both periventricular and juxtacortical distribution. Enhancement was not a prominent feature, suggesting that these lesions were likely chronic rather than acutely active. These findings suggested a demyelinating process (Figure 1). A neurology consultation was obtained the same day, and a provisional diagnosis of a motor and sensory relapse of relapsing-remitting MS (RRMS) was made, based on MRI findings consistent with demyelination. Full confirmation according to McDonald’s criteria, requiring evidence of dissemination in space and time (e.g., via CSF analysis and/or interval imaging), was pending at the time of discharge. Given the mild nature of the relapse and the patient’s preserved functional status, the potential use of corticosteroid therapy was considered. However, in line with current practice for mild relapses without significant functional impairment, corticosteroid therapy was not initiated. Cerebrospinal fluid (CSF) analysis was not performed during this presentation. He was discharged with an urgent referral to the MS multidisciplinary team (MDT) for outpatient management and long-term follow-up. 

Follow-up and clinical course

The patient’s initial NIHSS score in the ED was 2, reflecting mild but definite neurological impairment. He was discharged with an urgent referral to the MS multidisciplinary team for outpatient follow-up and interval imaging. While awaiting this, he re-attended the ED after sustaining a fall in the park while walking his dog, attributed to ongoing unsteadiness of gait. He was admitted to the medical ward for observation and underwent a repeat MRI, which demonstrated no new lesions or changes compared to the index scan. This confirmed clinical stability in the short term, despite the functional impact of his symptoms, and outpatient neurology follow-up was arranged to guide longer-term management.

## Discussion

The trap of diagnostic overshadowing 

This case underscores the significant risk of diagnostic overshadowing in patients with complex psychiatric histories. The initial presentation of left-sided numbness and unsteadiness, in the context of schizophrenia and learning disability, could easily have been attributed to a functional or psychogenic cause. Indeed, early documentation in the ED reflected this bias [1,4]. The overlap between MS and schizophrenia further complicates recognition, as both can manifest with cognitive impairment, psychosis, and a similar onset in young adults, increasing the risk of misdiagnosis. However, persistent and focal neurological signs, along with the collateral history of gradual decline, prompted further investigation. 

Differential diagnoses and clinical reasoning 

The differential diagnosis included ischemic stroke, which was initially prioritised due to vascular risk factors and focal signs. Functional neurological disorder was also considered, but it was less likely given the presence of objective neurological deficits [2,5]. His reported five-stone (approximately 32 kg) unintentional weight loss over the preceding six months was also noted, prompting consideration of alternative or concurrent causes such as malignancy, metabolic disorders, or chronic infection. However, the absence of systemic features and normal laboratory results made these unlikely. Antipsychotic therapy itself increases stroke and thromboembolic risk, underlining the importance of multidisciplinary vigilance in differentiating medication effects from new neurological disease. The CT head and CT angiogram were unremarkable. Ultimately, MRI imaging was decisive in revealing white matter changes consistent with demyelination. The provisional diagnosis of relapsing-remitting MS was supported by the distribution of lesions, which demonstrated dissemination in space. This case highlights the necessity of advanced imaging in the evaluation of subtle but progressive symptoms, especially when the initial CT is unremarkable [6]. 

Psychiatric comorbidity and diagnostic delay in MS 

In patients with psychiatric comorbidities, MS may be under-recognized or misdiagnosed. The literature has documented delays in diagnosis among this population, often due to the misattribution of neurological symptoms to mental illness [3,4]. A 2008 review by Jones et al. coined the term “diagnostic overshadowing” to describe this phenomenon, which has since been linked to poorer health outcomes and under-investigation in psychiatric patients [1,6]. Public-facing health resources also reflect the complexity of MS diagnosis, noting that misdiagnosis is not uncommon and often results from the broad and variable symptomatology of the condition [7]. Moreover, psychiatric comorbidity is common in MS itself, either preceding or resulting from disease onset. Neuropsychiatric symptoms are common in MS and may be under-reported or inadequately managed [5,8]. Anxiety, depression, and cognitive impairment can coexist with MS, further complicating diagnosis and management.

The value of family insights and MDT involvement 

In this case, the patient’s psychiatric history not only delayed initial escalation but also masked the progressive nature of his neurological symptoms. The detailed collateral account of progressive functional decline provided by his mother was pivotal in prompting escalation to MRI, even in the setting of unremarkable CT imaging and laboratory investigations. Furthermore, multidisciplinary input - including the stroke team, radiology, and neurology - was vital in achieving an accurate diagnosis and avoiding inappropriate discharge [9]. 

Key implications for ED practice

This case reinforces the need for emergency physicians to maintain a broad differential in patients with psychiatric comorbidities, to place significant weight on collateral history when initial investigations are unremarkable, and to escalate to advanced imaging such as MRI when clinical suspicion remains high. Early multidisciplinary collaboration can improve diagnostic accuracy, prevent premature closure, and ultimately lead to better patient outcomes.

## Conclusions

This case highlights the importance of maintaining diagnostic vigilance when evaluating neurological decline in patients with psychiatric comorbidities. Diagnostic overshadowing can obscure organic pathology and delay recognition of serious illness. In this instance, while departmental crowding contributed to delayed review, collateral history and recognition of progressive, unexplained symptoms prompted MRI, which was crucial for diagnosis. The case illustrates how psychiatric history should inform but never override clinical reasoning, and how early imaging in the presence of red flags can prevent premature closure. Family input and multidisciplinary collaboration remain essential, reinforcing that timely identification of concerning features can significantly alter outcomes in emergency care.
